# Ploidy as a prognostic indicator in end stage squamous cell carcinoma of the head and neck region treated with cisplatinum.

**DOI:** 10.1038/bjc.1990.169

**Published:** 1990-05

**Authors:** L. D. Cooke, T. G. Cooke, F. Bootz, G. Forster, T. R. Helliwell, D. Spiller, P. M. Stell

**Affiliations:** Department of Otorhinolaryngology, University of Liverpool, UK.

## Abstract

We measured tumour cellular DNA in 102 patients entered into two phase III trials of chemotherapy for end stage squamous carcinoma of the head and neck. The median survival of untreated patients with aneuploid tumours was 55 days compared with 224 days for patients treated with cisplatinum. This difference was highly significant. In contrast the median survival of untreated patients with diploid tumours was 74 days compared with 118 days for treated patients. Although this difference is statistically significant, the increased survival of 6 weeks is of no clinical benefit compared with the prolongation of survival of 6 months in patients with aneuploid tumours. Multivariate analysis showed that the significant predictors of survival were Karnofsky status, response to chemotherapy and ploidy.


Br. J. Cancer (1990), 61, 759-762 © Macmillan Press Ltd., 1990

### Ploidy as a prognostic indicator in end stage squamous cell carcinoma of the head and neck region treated with cisplatinum

L.D. Cooke[1], T.G. Cooke[2], F. Bootz[1], G. Forster[2], T.R. Helliwell[3], D. Spiller[4] & P.M. Stell[1]

*Departments of [1]Otorhinolaryngology, [2]Surgery, [3]Pathology and [4]Radiation Biology, University of Liverpool, Liverpool 69, UK.*

**Summary** We measured tumour cellular DNA in 102 patients entered into two phase III trials of chemotherapy for end stage squamous carcinoma of the head and neck. The median survival of untreated patients with aneuploid tumours was 55 days compared with 224 days for patients treated with cisplatinum. This difference was highly significant. In contrast the median survival of untreated patients with diploid tumours was 74 days compared with 118 days for treated patients. Although this difference is statistically significant, the increased survival of 6 weeks is of no clinical benefit compared with the prolongation of survival of 6 months in patients with aneuploid tumours. Multivariate analysis showed that the significant predictors of survival were Karnofsky status, response to chemotherapy and ploidy.

The relationship of ploidy to prognosis in solid tumours has been widely studied. In general patients with diploid tumours have been found to have a more favourable prognosis than those with aneuploid cancers (Cornelisse *et al.*, 1987; Joensuu *et al.*, 1986; Zimmerman *et al.*, 1987). Relatively few studies of squamous cell carcinoma of the head and neck have been reported and not all agree that aneuploidy confers a poor prognosis (Goldsmith *et al.*, 1987). However, recent studies of resectable squamous cell carcinoma suggest a poor outcome for those with an aneuploid DNA content (Kokal *et al.*, 1988).

There is preliminary evidence that DNA ploidy may also predict response to cytostatic therapy. In certain leukaemias treated by chemotherapy, the aneuploid tumours are the most responsive (Barlogie *et al.*, 1987). In order to test this hypothesis on solid tumours we have assessed the effect of ploidy in cancers from patients entered into two phase III prospective randomised trials of chemotherapy for end stage squamous carcinoma of the head and neck (Morton *et al.*, 1985; Allison *et al.*, 1989).

Entry into these trials was confined to patients with locally advanced or recurrent squamous cell cancers unsuitable for radiotherapy or surgery who were then randomised either to no further treatment or to various chemotherapy regimes. Of these, single agent cisplatinum produced the greatest prolongation of survival. Therefore in this investigation we have determined the tumour DNA content by flow cytometric analysis from patients in these trials who were randomised to receive cisplatinum or who acted as untreated controls.

### Patients and methods

### *Selection of patients*

Patients were recruited into two phase III trials of end stage squamous carcinoma of the head and neck between 1982 and 1987. A total of 315 patients with locally advanced or recurrent squamous cell cancer presenting to the Mersey Regional Head and Neck Unit were randomised prospectively to receive either chemotherapy or to act as untreated controls. For this investigation 88 patients randomised to receive cisplatinum and 95 who received no further treatment were selected. Only the 102 patients for whom archival paraffin embedded tissue from the last biopsy or operative specimen was available were studied.

Correspondence: L.D. Cooke, Department of Otorhinolaryngology, Glasgow Royal Infirmary, Glasgow G4 0SG, UK.
Received 25 October 1989; and in revised form 11 December 1989.

### *Dosage and administration*

Cisplatinum was administered as an infusion at a dosage of 100 mg m$^{-2}$ body surface area if the creatinine clearance exceeded 60 ml min$^{-1}$ and at half that dosage if the creatinine clearance lay between 50 and 60 ml min$^{-1}$. This treatment was given at monthly intervals until either the tumour progressed, toxicity became unacceptable or the patient refused further treatment.

### *Patient assessment*

The WHO definition of response of the tumour was used. A partial response was defined as a reduction of at least 50% in the product of two perpendicular diameters of all assessable lesions and a complete response was defined as the absence of clinically detectable disease (Miller *et al.*, 1981).

### *Flow cytometric analysis*

Thick sections were examined by flow cytometry. Consecutive 5 $\mu$m sections were stained by haematoxylin and eosin to confirm the presence of tumour in all samples studied. Nuclei were extracted from formalin fixed paraffin embedded tissue (Hedley *et al.*, 1983). Multiple 50 $\mu$m sections were dewaxed in xylene and rehydrated through 0.5% pepsin in 0.9% NaCl at pH 1.5 for 30 min at 37°C. The digest was then centrifuged, washed and resuspended. After resuspension in 1 ml of phosphate buffered saline the digest was syringed 3 or 4 times to disaggregate nuclear clumps and then filtered through 40 $\mu$m nylon mesh.

Nuclear concentrations were adjusted when necessary to give a final concentration of 10 nuclei per ml. DNA was analysed by a FACS flow cytometer (Becton Dickinson Sunnyvale, CA, USA). Where possible fluorescence from 100,000 nuclei was recorded, a minimum of 10,000 being required to give interpretable histograms. The use of paraffin sections containing normal cells acted as an internal control, enabling tumour cells with more or less than 2 c DNA to be detected. Histograms were classified as aneuploid or diploid, and only those with a coefficient of variation of less than 8% were accepted. Tetraploid tumours, especially if they represent a small fraction of the whole section may be difficult to detect as those tumours cells in the G0 and G1 phases of the cell cycle have the same DNA content as normal cells in G2 and M phase. In these cases a 4 c peak representing more than 20% of the whole cell population was designated aneuploid as it was unlikely that normal cells would have such a high G2/M peak.

*Statistical analysis*

Qualitative data such as response rates are displayed by contingency tables and analysed by the $\chi^2$ method. Survival rates are shown by the Kaplan-Meier product-limit estimate. Prognostic factors affecting survival were first identified by univariate methods using the log rank test (Peto, 1977) with analysis for trend where appropriate, and significant prognostic factors were then subjected to multivariate analysis using Cox's regression analysis.

*Staging and follow-up*

All the tumours were classified by the latest TMN scheme (UICC, 1988) and the patients performance status recorded by Karnofsky's method. Histological assessment of the degree of tumour differentiation was performed by one pathologist (TRH). The patients have all been followed up until death or within 6 weeks of the report if they are still alive (median survival of 125 days). No patient has been lost to follow-up.

### Results

*Ploidy*

The coefficient of variation varied between 3.2 and 7.9 (median 6.4). Forty-seven (46%) of the tumours were classified as diploid and 55 (54%) aneuploid. Of the 52 tumours from patients who received cisplatinum, 22 (42%) were diploid and 30 (58%) were aneuploid. In the untreated group 25 tumours (50%) were diploid and 25 (50%) were aneuploid. The relationships between DNA content and sex, age, performance status, site of the primary tumour, stage grouping, histological grade and previous treatment are shown in Tables I and II. There was no significant relationship between any of these parameters and ploidy.

*Chemotherapy*

The mean number of courses of cisplatinum received by the group of patients with diploid tumours was 2.5 and that by the group with aneuploid tumours was 2.6

*Relationship of ploidy to response*

Twenty-two (40%) of the treated patients had a partial or complete response to cisplatinum. Of the 22 diploid tumours, eight (36%) patients showed a partial or complete response compared with 14 of 30 (47%) aneuploid tumours. This difference was not significant ($\chi^2 = 2.92$) (Table III).

*Relationship of ploidy to survival*

The median survival of untreated patients with diploid tumours was 74 days and of those with aneuploid tumours 55 days, but this difference did not reach statistical significance ($\chi^2 = 1.72$) (Figure 1). The median survival of treated patients with aneuploid tumours was 224 days and the increased survival of this treated group was significantly longer than that for patients with untreated aneuloid cancers ($\chi^2 = 20.7$, $P < 0.001$) (Figure 2). The median survival of treated patients with diploid tumours was 118 days and this was significantly longer than the median survival of untreated patients with diploid tumours ($\chi^2 = 7.31$, $P < 0.01$) (Figure 3).

*Prognostic factors*

Cox's regression analysis showed that age, sex, site of disease, histological grading and previous treatment were not significant prognostic factors. Karnofsky status and response to chemotherapy were both highly significant predictors of survival ($P < 0.001$). An aneuploid tumour DNA content was also found to be a significant predictor of survival ($P < 0.025$). Stage was not included in this analysis as most tumours were in stage IV.

**Table I** Patient details: host factors

| | *Untreated* | | *Treated* | |
|---|---|---|---|---|
| | *Diploid* | *Aneuploid* | *Diploid* | *Aneuploid* |
| Age (mean) | 61.8 | 68.0 | 60.8 | 62.5 |
| Sex M | 15 | 16 | 16 | 22 |
| F | 10 | 9 | 6 | 8 |
| Karnofsky status | | | | |
| Median | 60 | 60 | 60 | 70 |
| Range | 20-90 | 20-80 | 30-80 | 40-70 |
| Previous treatment | | | | |
| None | 4 | 6 | 8 | 7 |
| DXT/surgery | 21 | 19 | 16 | 23 |

**Table II** Patients details: tumour factors

| | *Untreated* | | *Treated* | |
|---|---|---|---|---|
| | *Diploid* | *Aneuploid* | *Diploid* | *Aneuploid* |
| Site | | | | |
| Mouth | 3 | 10 | 5 | 7 |
| Oropharynx | 7 | 3 | 5 | 3 |
| Hypopharynx | 10 | 5 | 6 | 7 |
| Larynx | 1 | 5 | 5 | 9 |
| Other | 4 | 2 | 1 | 4 |
| Disease stage | | | | |
| I | 1 | 0 | 0 | 0 |
| II | 3 | 0 | 0 | 2 |
| III | 3 | 6 | 1 | 2 |
| IV | 18 | 19 | 21 | 26 |
| Histological grade | | | | |
| Poor | 12 | 14 | 14 | 20 |
| Moderate | 10 | 7 | 4 | 7 |
| Well | 3 | 4 | 4 | 3 |

**Table III** Response to treatment

| | *Progression* | *No change* | *Partial response* | *Complete response* |
|---|---|---|---|---|
| Diploid | 10 | 4 | 7 | 1 |
| Aneuploid | 7 | 9 | 13 | 1 |

**Figure 1** Survival of untreated patients with diploid and aneuploid tumours.

**Figure 2** Survival of treated and untreated patients with aneuploid tumours.

**Figure 3** Survival of treated and untreated patients with diploid tumours.

### Discussion

Analysis of DNA content can provide important prognostic information in a variety of malignant neoplasms. Abnormal cellular DNA contents clearly worsen the prognosis in breast carcinoma (Cornlisse *et al.*, 1987), bronchial carcinoma (Zimmerman *et al.*, 1987), colorectal carcinoma (Armitage *et al.*, 1985), prostatic carcinoma (Fordham *et al.*, 1986) and squamous cell carcinoma at sites outside the head and neck, such as the lung (Blondal, 1981) and cervix (Jakobsen, 1984).

Many of the increasing number of studies of squamous cell carcinoma of the upper aerodigestive tract attempt only to relate DNA content to clinical and pathological tumour parameters, rather than to clinical outcome. In general there has been no consistent relationship between ploidy or proliferative characteristics and either pathological grade or clinical stage.

No relationship was shown between ploidy and histological grade by Kaplan *et al.* (1986), Johnson *et al.* (1985) and Ensley *et al.* (1989) but the frequency of DNA non-diploid tumours was found to correlate with a decrease in the histological grading by Holm (1982), Tytor *et al.* (1987) and Feichter *et al.* (1987), who also found that it was directly proportional to the percentage of cells in S-phase. We found no correlation between the degree of histological differentiation and DNA content. However, a parallel study of the histopathology of these carcinomas has revealed other morphological features which correlate with ploidy, diploid carcinomas having prominent nucleoli and a lower surface area to volume ratio of the groups of tumour cells (Helliwell *et al.*, 1989).

No significant relationship between ploidy and tumour stage was found by Feichter *et al.* (1987), Holm (1982) and Ensley *et al.* (1989), whereas Kaplan *et al.* (1986), Kokal *et al.* (1988) and Tytor *et al.* (1989) reported a higher incidence of aneuploidy in advanced tumours.

Data relating clinical outcome to DNA content in larger series of patients treated in a uniform manner are few. Kokal *et al.* (1988) studied a group of 76 patients with surgically resectable lesions of the oral cavity, larynx and pharynx. Those patients with aneuploid cancers had significantly lower relapse-free and overall survival rates, and Cox's regression analysis showed tumour DNA content to be an independent prognostic factor. In surgically treated squamous cell carcinoma of the oesophagus a high hyperploid DNA content was an independent prognostic factor indicating a poor prognosis (Matsuura *et al.*, 1986).

In a large series of oral cavity carcinomas (Tytor *et al.*, 1989), stage I and II aneuploid tumours had a worse prognosis, but the reverse was true for stages III and IV which were mostly treated by combined surgery and radiotherapy or radiotherapy alone. A previous observation that aneuploid tumours responded better to preoperative radiotherapy (Franzen *et al.*, 1986) was further confirmed in this later study, and may account for the reversal of the prognosis for those with stage III and IV aneuploid tumours. This finding may also explain the report by Goldsmith *et al.* (1986) that, among a heterogeneous group of patients with laryngeal carcinoma, the aneuploid tumours had a better prognosis as all but five of the 48 patients received radiotherapy as part of their treatment. Franzen *et al.* (1986) also showed that the mean S-phase value was higher (16.1%) in those tumours that were eradicated by preoperative radiotherapy than for those that did not respond (8.1%).

A strong direct correlation between the degree of DNA aneuploidy and S-phase fraction has been reported for squamous carcinomas of the head and neck (Ensley *et al.*, 1989) as well as a number of different solid tumours (Barlogie *et al.*, 1983). This suggests that aneuploid tumours may be faster growing and therefore more susceptible to chemotherapy. In certain haematological malignancies treated by chemotherapy, such as adults with acute myelogenous leukaemia and children with acute lymphocytic leukaemia, aneuploidy has emerged as a favourable prognostic factor (Barlogie *et al.*, 1987; Look *et al.*, 1984). Similarly hyperdiploid neuroblastomas in infants responded better to chemotherapy than diploid tumours (Look *et al.*, 1985).

Our results show a slightly longer median survival for patients with diploid tumours compared with those with aneuploid tumours receiving no further treatment. Paradoxically, response rates did not differ significantly between diploid and aneuploid tumours but survival did. This is further evidence of the unreliability of response in assessing the efficacy of chemotherapy.

Diploid tumours may be less susceptible to cytotoxic therapy, either because fewer cells are undergoing DNA synthesis, or because of undefined characteristics confering resistence that are independent of kinetic considerations.

Our data suggest that in end stage squamous cell carcinoma of the head and neck the aneuploid tumours are most responsive to chemotherapy. In this study chemotherapy increased survival for patients with diploid cancers by only 6 weeks, compared with an increase of 6 months for those with aneuploid tumours. It is therefore doubtful whether cytotoxic therapy for patients with diploid cancers is worthwhile. As only 30–40% of these tumours responded to either single agent or combination regimes, evaluation of DNA content may aid in the selection of those patients who might benefit.

### References

ALLISON, R.S., CAMPBELL, J.B., DALBY, J.B. & 8 others (1990). A phase III randomised trial of cisplatinum, methotrexate, cisplatin + methotrexate and cisplatinum + 5-FU in end stage squamous carcinoma of the head and neck. *Br. J. Cancer* (in the press).

ARMITAGE, N.C., ROBIAS, R.A., EVANS, D.F., *et al.* (1985). The influence of tumour cell DNA abnormalities on survival in colorectal cancer. *Br. J. Surg.*, **72**, 828.

BARLOGIE, B., RABER, M.N., SCHUMANN, J. & 6 others (1983). Flow cytometry in clinical cancer research. *Cancer Res.*, **43**, 3982.

BARLOGIE, B., STASS, S., DIXON, D. & 5 others (1987). DNA aneuploidy in adult acute leukaemia. *Cancer Genet. Cytogenet.*, **28**, 213.

BLONDAL, T. & BENGTSSEN, A. (1981). Nuclear DNA measurements in squamous cell carcinoma of the lung: a guide for prognostic evaluation. *Anticancer Res.*, **1**, 79.

CAMPBELL, J.B., DORMAN, E.B., HELLIWELL, T.R. & 8 others (1987). Factors predicting response of end stage squamous cell carcinoma of the head and neck to cisplatinum. *Clin. Otolaryngol.*, **12**, 167.

CORNELISSE, C.J., VAN DE VELDE, C.J.H., CASPERS, R.J.C., MOOLENAR, A.J. & HERMANS, J. (1987). DNA ploidy and survival in breast cancer patients. *Cytometry*, **8**, 225.

ENSLEY, J.F., MACIOROWSKI, Z., HASSAN, M. & 6 others (1989). Cellular DNA content parameters in untreated and recurrent squamous cell cancers of the head and neck. *Cytometry*, **10**, 334.

FEICHTER, G.E., MAIER, H., ADLER, D. & 4 others (1987). S-phase fractions and DNA ploidy of oropharyngeal squamous epithelium carcinomas compared with histological grade, stage, response to chemotherapy and survival. *Acta Otolaryngol.*, **104**, 377.

FORDHAM, M.V.P., BURDE, A.H., MATHEWS, J., WILLIAMS, G. & COOKE, T.G. (1986). Prostatic carcinoma cell DNA content measured by flow cytometry and its relation to clinical outcome. *Br. J. Surg.*, **73**, 400.

FRAZEN, G., KLINTENBERG, C., OLOFSSON, J. & RISBERG, B. (1986). DNA measurement – an objective predictor of response to irradiation? A review of 24 squamous cell carcinomas of the oral cavity. *Br. J. Cancer*, **53**, 643.

GOLDSMITH, M.G., CREESON, D.H., ARNOLD, L.A., POSTMA, D.S., ASKIN, F.B. & PILLSBURY, H.C. (1987). DNA flow cytometry as a prognostic indicator in head and neck cancer. *Otolaryngol. Head Neck Surg.*, **96**, 307.

HEDLEY, D., FRIEDLANDER, M., TAYLOR, I., RUGG, C. & MUSGROVE, E. (1983). Method of analysis of cellular DNA content of paraffin embedded pathological material using flow cytometry. *J. Histochem. Cytochem.*, **31**, 1333.

HELLIWELL, T.R., ATKINSON, M.W., COOKE, L.D., COOKE, T.G. & STELL, P.M. (1990). Morphometric analysis, ploidy and response to chemotherapy in squamous carcinomas of the head and neck. *Pathol. Res. Prac.* (in the press).

HOLM, L.E. (1982). Cellular DNA amounts of squamous cell carcinoma of the head and neck region in relation to prognosis. *Laryngoscope*, **92**, 1064.

HOLM, L.E, JAKOBSSON, P., KILLANDER, B., SILFVERSWARD, C. & WERSALL, J. (1980). DNA and its synthesis in individual tumour cells from human upper respiratory tract squamous cell carcinomas. *Laryngoscope*, **90**, 1209.

JAKOBSEN, A. (1984). Prognostic impact of ploidy level in carcinoma of the cervix. *Am. J. Clin. Oncol.*, **7**, 475.

JOENSUU, H., KLEMI, P., EEROLA, E. & TUOMINEN, J. (1986). Influence of cellular DNA content on survival in differentiated thyroid carcinoma. *Cancer*, **58**, 2462.

JOHNSON, T.S., WILLIAMSON, K.D., CRAMER, M.M. & PETERS, L.J. (1985). Flow cytometric analysis of head and neck carcinoma DNA index and S-phase fraction from paraffin-embedded sections: comparison with malignancy grading. *Cytometry*, **6**, 461.

KAPLAN, A.S., CALDARELLI, D.D, CHACHO, M.S. & 4 others (1986). Retrospective DNA analysis of head and neck squamous cell carcinoma. *Arch. Otolaryngol. Head Neck Surg.*, **112**, 1159.

KOKAL, A.K., GARDINE, R.L., SHEIBANI, K. & 5 others (1988). Tumour DNA content as a prognostic indicator in squamous cell carcinoma of the head and neck region. *Am. J. Surg.*, **156**, 276.

LOOK, A.T., HAYES, F.A., NITSCHKE, R., MCWILLIAMS, M.B. & GREEN, A.A. (1984). Cellular DNA content predicts response to cylclophosphamide and doxorubicin in infants with unresectable neuroblastoma. *N. Engl. J. Med.*, **311**, 231.

LOOK, A.T., ROBERSON, P.K., WILLIAMS, D.L. & 9 others (1985). Prognostic importance of blast cell content in childhood acute lymphoblastic leukemia. *Blood*, **65**, 1079.

MATSUURA, H., SUGIMACHI, K., UEO, H., KUWANO, H., KOGA, Y. & OKAMURA, T. (1986). Malignant potentiality of squamous cell carcinoma of the oesophagus predictable by DNA analysis. *Cancer*, **57**, 1810.

MILLER, A.B., HOOGSTRATEN, B., STAQUET, M. & WINKLER, A. (1981). Reporting results of cancer treatment. *Cancer*, **47**, 207.

MORTON, R.P., RUGMAN, F., DORMAN, E.B. & 5 others (1985). Cisplatinum and Bleomycin for advanced or recurrent squamous cell carcinoma of the head and neck: a randomised factorial phase III controlled trial. *Cancer Chemother. Pharmacol.*, **15**, 283.

PETO, R., PIKE, M.C, ARMITAGE, P. & 7 others (1977). Design and analysis of randomised clinical trials requiring prolonged observation of each patient. *Br. J. Cancer*, **35**, 1.

TYTOR, M., FRANZEN, G. & OLOFSSON, J. (1987). DNA pattern in oral cavity carcinomas in relation to clinical stage and histological grading. *Pathol. Res. Pract.*, **182**, 202.

TYTOR, M., FRANZEN, G. & OLOFSSON, J. (1989). DNA ploidy in oral cavity carcinomas with special reference to prognosis. *Head Neck*, **11**, 257.

ZIMMERMAN, P.V., HAWSON, G.A.T., BINT, M.H. & PARSONS, P.G. (1987). Ploidy as a prognostic determinant in surgically treated lung cancer. *Lancet*, **ii**, 530.


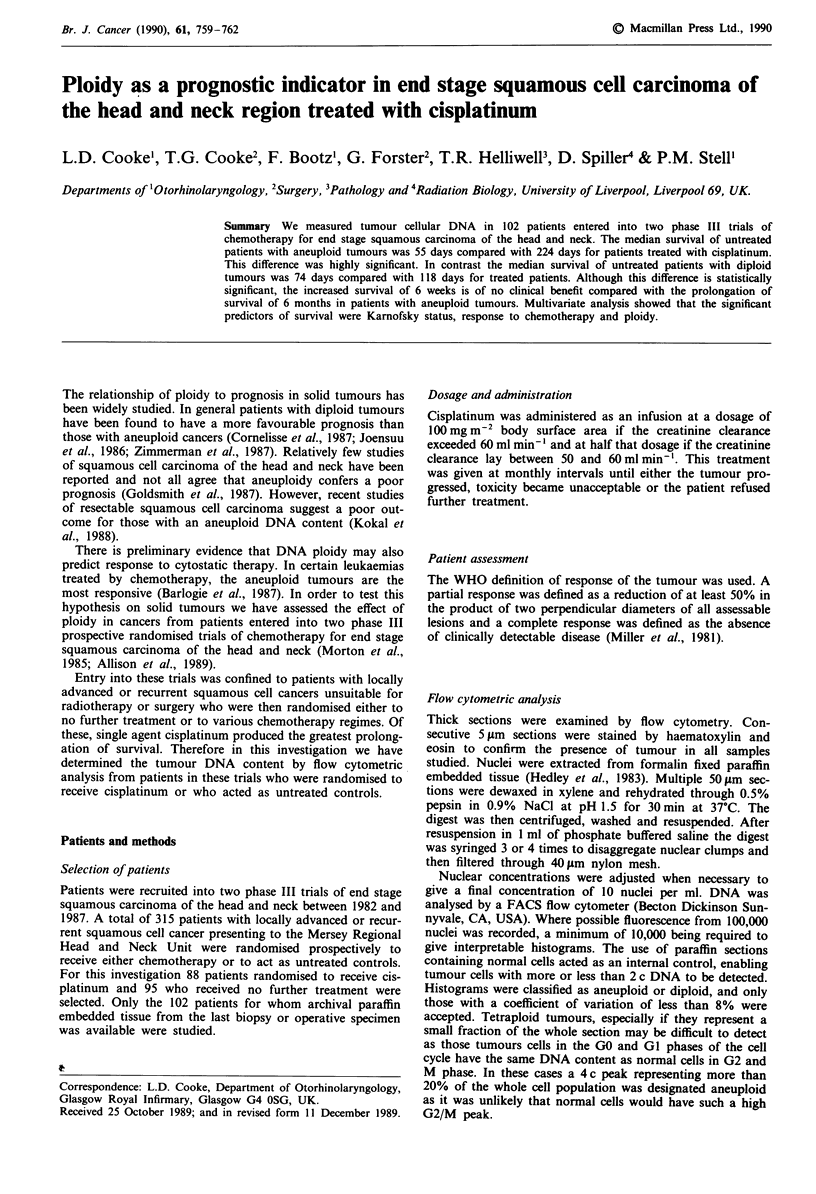

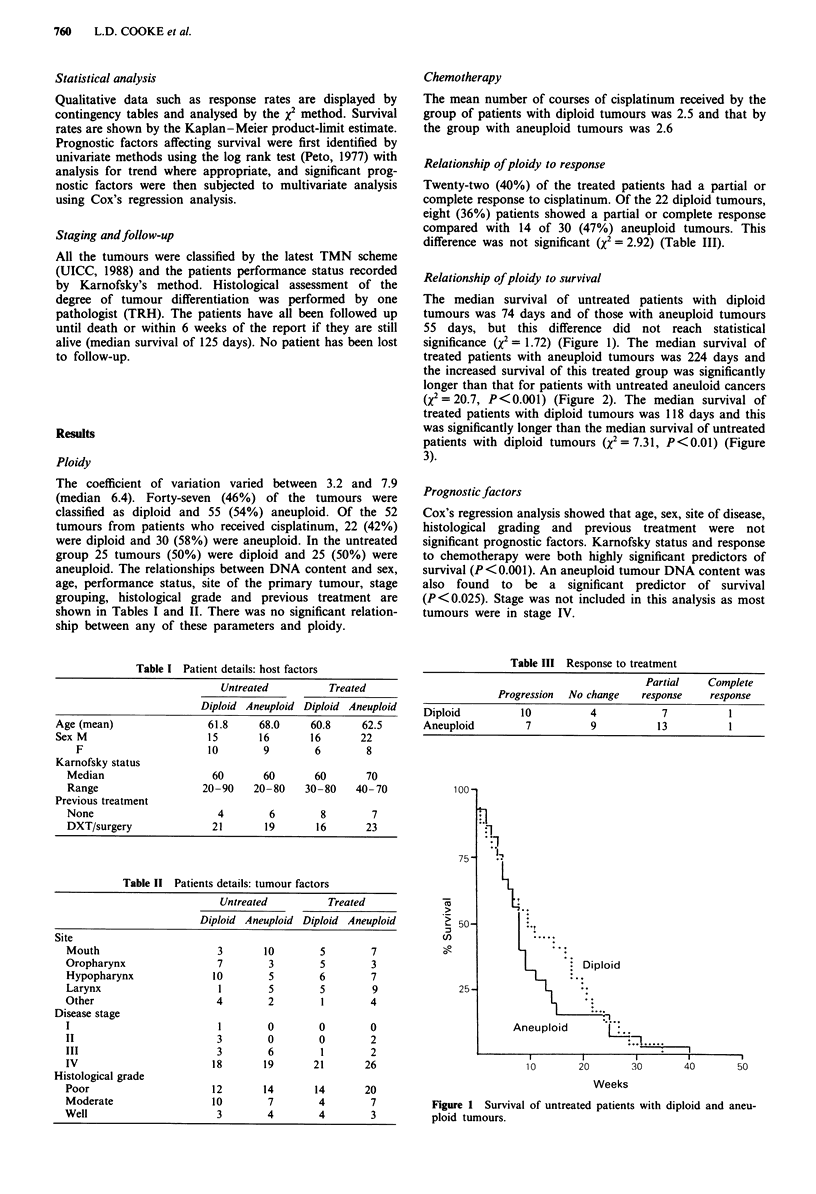

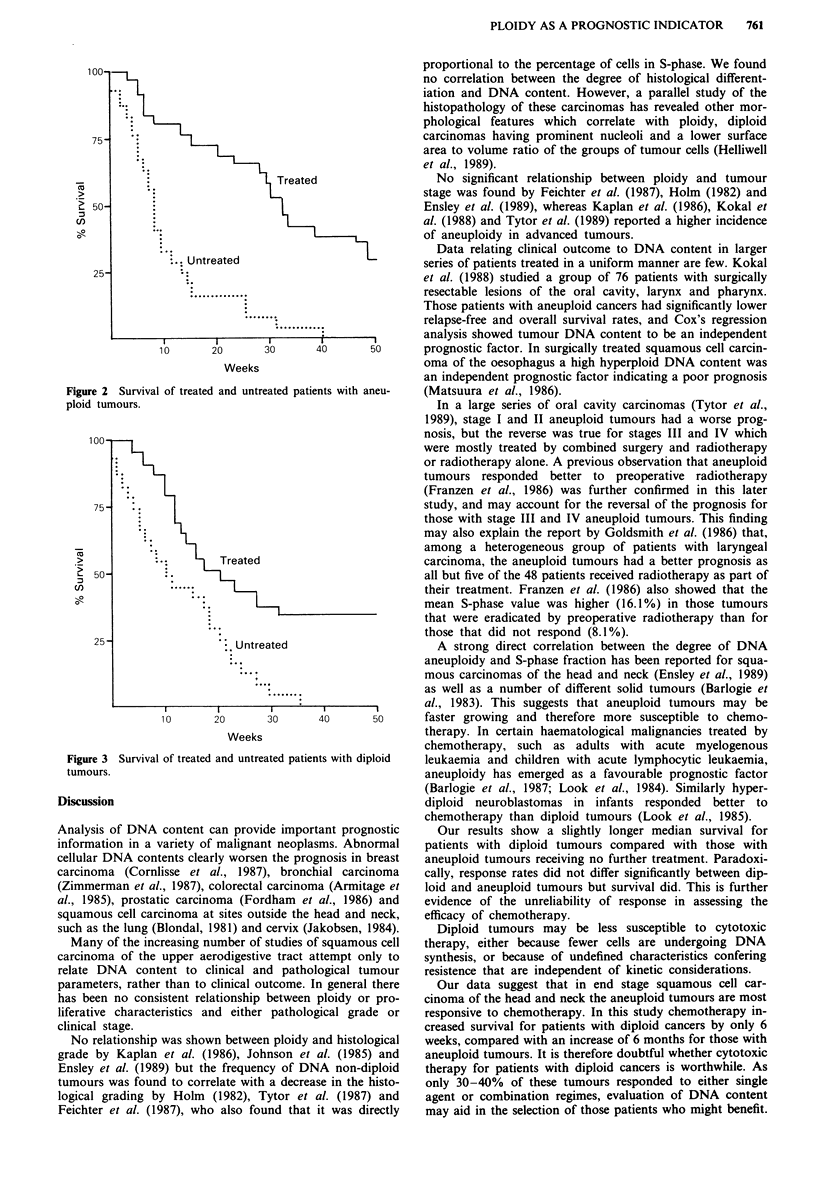

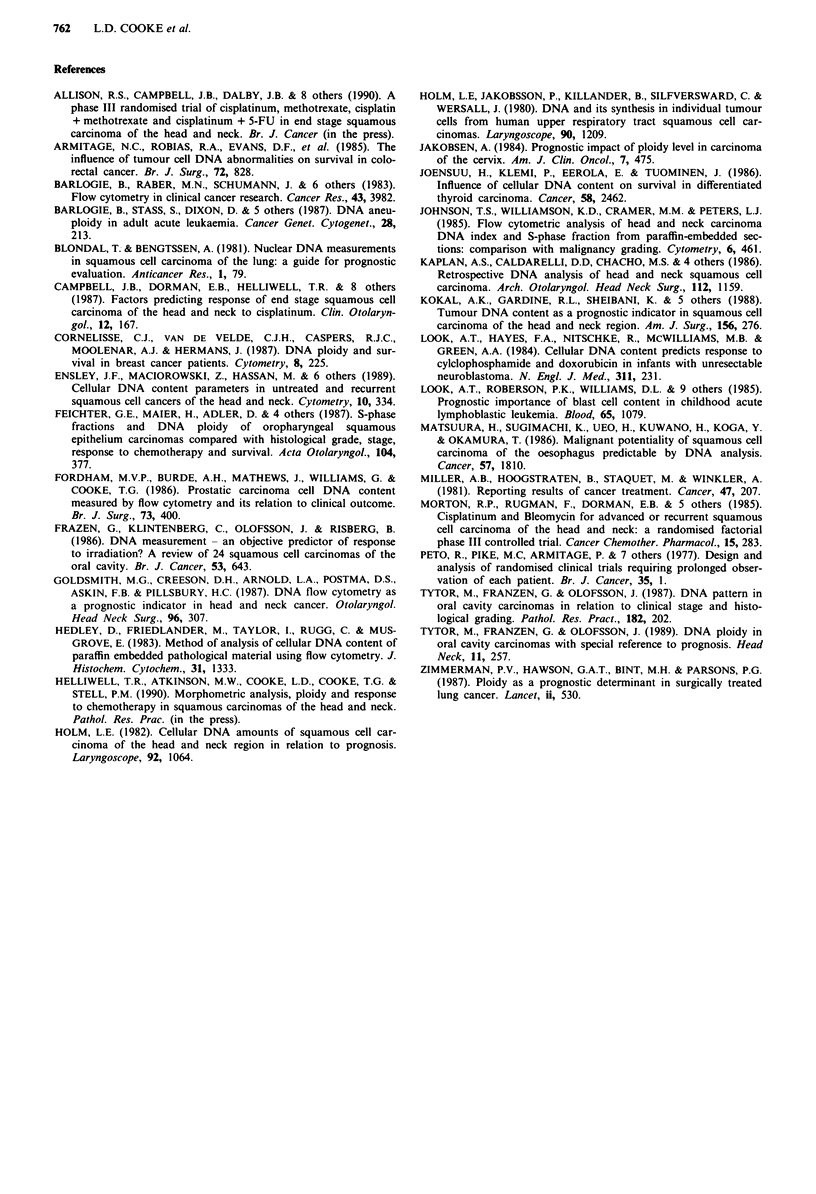


## References

[OCR_00427] Armitage N. C., Robins R. A., Evans D. F., Turner D. R., Baldwin R. W., Hardcastle J. D. (1985). The influence of tumour cell DNA abnormalities on survival in colorectal cancer.. Br J Surg.

[OCR_00434] Barlogie B., Raber M. N., Schumann J., Johnson T. S., Drewinko B., Swartzendruber D. E., Göhde W., Andreeff M., Freireich E. J. (1983). Flow cytometry in clinical cancer research.. Cancer Res.

[OCR_00435] Barlogie B., Stass S., Dixon D., Keating M., Cork A., Trujillo J. M., McCredie K. B., Freireich E. J. (1987). DNA aneuploidy in adult acute leukemia.. Cancer Genet Cytogenet.

[OCR_00445] Campbell J. B., Dorman E. B., Helliwell T. R., McCormick M., Miles J., Morton R. P., Rugman F., Stell P. M., Stoney P. J., Vauhan E. D. (1987). Factors predicting response of end stage squamous cell carcinoma of the head and neck to cisplatinum.. Clin Otolaryngol Allied Sci.

[OCR_00451] Cornelisse C. J., van de Velde C. J., Caspers R. J., Moolenaar A. J., Hermans J. (1987). DNA ploidy and survival in breast cancer patients.. Cytometry.

[OCR_00456] Ensley J. F., Maciorowski Z., Hassan M., Pietraszkiewicz H., Heilbrun L., Kish J. A., Tapazoglou E., Jacobs J. R., al-Sarraf M. (1989). Cellular DNA content parameters in untreated and recurrent squamous cell cancers of the head and neck.. Cytometry.

[OCR_00460] Feichter G. E., Maier H., Adler D., Born I. A., Abel U., Haag D., Goerttler K. (1987). S-phase fractions and DNA-ploidy of oropharyngeal squamous epithelium carcinomas compared with histologic grade, stage, response to chemotherapy and survival.. Acta Otolaryngol.

[OCR_00467] Fordham M. V., Burdge A. H., Matthews J., Williams G., Cooke T. (1986). Prostatic carcinoma cell DNA content measured by flow cytometry and its relation to clinical outcome.. Br J Surg.

[OCR_00473] Franzén G., Klintenberg C., Olofsson J., Risberg B. (1986). DNA measurement--an objective predictor of response to irradiation? A review of 24 squamous cell carcinomas of the oral cavity.. Br J Cancer.

[OCR_00479] Goldsmith M. M., Cresson D. H., Arnold L. A., Postma D. S., Askin F. B., Pillsbury H. C. (1987). DNA flow cytometry as a prognostic indicator in head and neck cancer.. Otolaryngol Head Neck Surg.

[OCR_00487] Hedley D. W., Friedlander M. L., Taylor I. W., Rugg C. A., Musgrove E. A. (1983). Method for analysis of cellular DNA content of paraffin-embedded pathological material using flow cytometry.. J Histochem Cytochem.

[OCR_00497] Holm L. E. (1982). Cellular DNA amounts of squamous cell carcinomas of the head and neck region in relation to prognosis.. Laryngoscope.

[OCR_00502] Holm L. E., Jakobsson P., Killander D., Silfverswärd C., Wersäll J. (1980). DNA and its synthesis in individual tumor cells from human upper respiratory tract squamous cell carcinomas.. Laryngoscope.

[OCR_00508] Jakobsen A. (1984). Prognostic impact of ploidy level in carcinoma of the cervix.. Am J Clin Oncol.

[OCR_00512] Joensuu H., Klemi P., Eerola E., Tuominen J. (1986). Influence of cellular DNA content on survival in differentiated thyroid cancer.. Cancer.

[OCR_00517] Johnson T. S., Williamson K. D., Cramer M. M., Peters L. J. (1985). Flow cytometric analysis of head and neck carcinoma DNA index and S-fraction from paraffin-embedded sections: comparison with malignancy grading.. Cytometry.

[OCR_00522] Kaplan A. S., Caldarelli D. D., Chacho M. S., Bruce D. R., Hutchinson J., Conway S., Coon J. S. (1986). Retrospective DNA analysis of head and neck squamous cell carcinoma.. Arch Otolaryngol Head Neck Surg.

[OCR_00527] Kokal W. A., Gardine R. L., Sheibani K., Zak I. W., Beatty J. D., Riihimaki D. U., Wagman L. D., Terz J. J. (1988). Tumor DNA content as a prognostic indicator in squamous cell carcinoma of the head and neck region.. Am J Surg.

[OCR_00531] Look A. T., Hayes F. A., Nitschke R., McWilliams N. B., Green A. A. (1984). Cellular DNA content as a predictor of response to chemotherapy in infants with unresectable neuroblastoma.. N Engl J Med.

[OCR_00537] Look A. T., Roberson P. K., Williams D. L., Rivera G., Bowman W. P., Pui C. H., Ochs J., Abromowitch M., Kalwinsky D., Dahl G. V. (1985). Prognostic importance of blast cell DNA content in childhood acute lymphoblastic leukemia.. Blood.

[OCR_00542] Matsuura H., Sugimachi K., Ueo H., Kuwano H., Koga Y., Okamura T. (1986). Malignant potentiality of squamous cell carcinoma of the esophagus predictable by DNA analysis.. Cancer.

[OCR_00548] Miller A. B., Hoogstraten B., Staquet M., Winkler A. (1981). Reporting results of cancer treatment.. Cancer.

[OCR_00551] Morton R. P., Rugman F., Dorman E. B., Stoney P. J., Wilson J. A., McCormick M., Veevers A., Stell P. M. (1985). Cisplatinum and bleomycin for advanced or recurrent squamous cell carcinoma of the head and neck: a randomised factorial phase III controlled trial.. Cancer Chemother Pharmacol.

[OCR_00561] Tytor M., Franzén G., Olofsson J. (1987). DNA pattern in oral cavity carcinomas in relation to clinical stage and histological grading.. Pathol Res Pract.

[OCR_00566] Tytor M., Franzén G., Olofsson J. (1989). DNA ploidy in oral cavity carcinomas, with special reference to prognosis.. Head Neck.

[OCR_00571] Zimmerman P. V., Hawson G. A., Bint M. H., Parsons P. G. (1987). Ploidy as a prognostic determinant in surgically treated lung cancer.. Lancet.

